# Patient and community experiences of tuberculosis diagnosis and care within a community-based intervention in Ethiopia: a qualitative study

**DOI:** 10.1186/s12889-015-1523-x

**Published:** 2015-02-25

**Authors:** Olivia Tulloch, Sally Theobald, Fukushi Morishita, Daniel G Datiko, Girum Asnake, Tadesse Tesema, Habiba Jamal, Paulos Markos, Luis E Cuevas, Mohammed A Yassin

**Affiliations:** Department of International Public Health, Liverpool School of Tropical Medicine, Pembroke Place, Liverpool, L3 5QA UK; HHA/REACH ETHIOPIA, Hawassa, SNPPR Ethiopia; Department of Clinical Sciences, Liverpool School of Tropical Medicine, Pembroke Place, Liverpool, L3 5QA UK; Global Fund to Fight AIDS, Tuberculosis and Malaria, Chemin des Blandonnet 8, 1214 Vernier, Geneva Switzerland

**Keywords:** Qualitative, TB, Community perspectives, Health extension workers, Close-to-community

## Abstract

**Background:**

The Ethiopian TB control programme relies on passive case finding of TB cases. The predominantly rural-based population in Ethiopia has limited access to health facilities creating barriers to TB services. An intervention package aimed to bring TB diagnosis and treatment services closer to communities has been implemented through partnership with health extension workers (HEWs). They undertook advocacy, communication and social mobilization (ACSM) activities, identified symptomatic individuals, collected sputum, prepared smears and fixed slides at community level. Field supervisors supported HEWs by delivering smeared slides to the laboratory, feeding back results to the HEWs and following up smear-negative cases. Patients diagnosed with TB initiated treatment in the community, they were supported by supervisors and HEWs through the local health post. Case notification increased from 64 to 127/100,000 population/year.

**Methods:**

This qualitative study assessed community members’ treatment seeking behaviour and their perceptions of the intervention. In-depth interviews (n=36) were undertaken with participants in six districts. Participants were clients of the community-based intervention, currently on TB treatment or those screened negative for TB. Transcripts were translated to English and a thematic analytical framework was developed guided by the different steps symptomatic individuals take within the intervention package. Coding was done and queries run using NVivo software.

**Results:**

Prior to the intervention many patients with chronic cough did not access TB services. Participants described difficulties they faced in accessing district level health facilities that required travel outside their communities. Giving sputum samples and receiving results from within their home communities was appreciated by all participants. The intervention had a high level of acceptability; particularly clear benefits emerged for poor women and men and those too weak to travel. Some participants appeared to prefer a diagnosis of TB, this is likely because receiving a negative smear microscopy result brought further uncertainty and necessitated seeking further investigation.

**Conclusions:**

There is evidence rural populations with high levels of poverty, and in particular women, are at high risk of unmet health needs and undiagnosed TB. Embedding TB services within communities was an acceptable approach for vulnerable groups experiencing poor access to health facilities. In the Ethiopian context this approach can facilitate early diagnosis and improve treatment outcomes.

## Background

In 2009 the World Health Organisation urged member states to move urgently towards universal access to tuberculosis (TB) prevention, early diagnosis and proper treatment, to the maximum extent possible [1]. In addition it recommends strengthening surveillance and improving diagnostic capacity in order to improve case detection [2]. The poor experience disproportionate barriers in accessing TB diagnostic services and can incur the highest relative costs relating to illness and healthcare [3-5]. These are important causes of delay in accessing TB services and poor women have been identified as particularly vulnerable [6]. Enhancing access to TB diagnosis and treatment to these most vulnerable groups is essential [7,8].

Active case finding (ACF) is a screening strategy used to systematically search for possible TB cases in groups thought to be at high risk, rather than waiting for patients to present themselves for medical attention. Screening aims to ensure that active TB is detected early and treatment is initiated promptly and so reduce poor disease outcomes and the adverse social and economic consequences of TB [9]. ACF is increasingly used alongside efforts to improve advocacy, communication and social mobilization for TB and other diseases. There are a growing number of innovative approaches aimed at increasing case finding that include using informal private providers [10-13] and community health workers [14,15]. Two systematic reviews suggest that while TB ACF is efficient in key risk groups and high burden communities, there is limited objective evidence available: in these reviews the proportion of invited persons who consented to undergo TB screening was used as a quantitative proxy (percentage screened among number eligible) for the acceptability of TB screening yet few studies take into account the qualitative perceptions of those being screened [16,17]. Acceptability is a social construct, yet community perspectives on acceptability of ACF are not sufficiently considered; qualitative data can bring insights to help to legitimize or improve screening processes for all those targeted.

TB is one of the major causes of morbidity and mortality in Ethiopia, a country with the seventh highest TB burden in the world. The national TB programme (NTP) relies on passive case finding at health facilities, but there is a recognized need to strengthen community screening [18]. About 84% of Ethiopians live in rural areas [19] and difficult terrain and poor road infrastructure hinders access to health facilities. Diagnostic delays also occur due to stigma and lack of knowledge, poverty, reliance on traditional healers and the need for repeated visits to healthcare facilities to achieve a correct diagnosis [20-23]. Non-educated individuals and women have been identified as needing targeted interventions to improve TB health seeking behaviour [24].

In 2003 Ethiopia launched a Health Extension Programme (HEP) to provide community-based health interventions. The programme aims to promote equity through improved access to preventive essential health interventions and increased health awareness [25]. Two salaried, female health extension workers (HEWs) provide services in each *kebele* (the lowest administrative unit with average population of about 5000 people). HEWs are stationed at health posts within their communities, provide health promotion at the household level and are the first point of contact for the community to the health system [26]. Volunteer community health promoters (CHPs) - who were subsumed into a ‘health development army’ in 2013 - aid HEWs in activities at household level. The HEWs provide services from sixteen health ‘packages’, one of which is TB prevention and control. The TB package in the HEP however is limited to awareness creation, referral of symptomatic individuals to diagnostic facilities and supporting treatment adherence.

Here we report on a study exploring the community perspectives of a community-based TB intervention package in Southern Ethiopia. Prior to the introduction of the intervention package health facilities used passive case finding and the HEP identified individuals with cough symptoms who were referred them to health facilities. Data for the project zone showed that in the previous 5 years case detection had not increased, and during the intervention did increase. Results of the improved case detection and treatment outcome resulting from the intervention were published elsewhere: HEWs screened 49,857 symptomatic individuals (60% women) in one year; 2,262 (4.5%) had smear-positive TB (53% women) and case notification increased from 64 to 127/100,000 population [27]. There is a need document the experiences and perceptions of the vulnerable groups targeted by the intervention package, including those who received a smear negative result, as these are not well understood. In addition, we seek to respond to the recognized gap in exploring acceptability of such TB interventions qualitatively. This evidence is important to ensure a human rights’-based approach is employed [9] and to inform plans for scale up and sustainability of the interventions in Ethiopia.

## Methods

Since 2010 the project has worked in collaboration with the Sidama Zone Health Department (Southern Ethiopia) and the NTP to undertake a community-based TB intervention package in partnership with HEWs. The role of the HEWs and CHPs was expanded to include advocacy, communication and social mobilization (ACSM) activities, identifying symptomatic individuals, collecting sputum and preparing smears at community level. Sputum collection occurred either at in people’s homes during house-to-house visits, or at the health post, depending on where screening was taking place. TB advocacy and house-to-house visits for all households within the *kebele* are a routine part of the existing HEP, but active TB screening, diagnostic and treatment activities were innovations in this intervention. Information about TB symptoms and the availability of the intervention’s services was delivered through local radio, religious and social gatherings, *kebele* health posts, house-to-house visits and community gatherings. Field supervisors supported HEWs by delivering smeared slides to the laboratory, feeding back results to the HEWs, initiating treatment, conducting contact tracing and following up smear-negative cases. Patients diagnosed with TB initiated treatment in the community, supported by supervisors and HEWs and organized through the local health post, those who received a smear negative result were visited and requested to resubmit sputum samples or referred for further examination as necessary, as described in Yassin et al. [27].

This study used qualitative methods to explore community experiences of the range of services delivered through the project, to understand the context and gain insight from the target populations [28]. Participants were screened and tested via the intervention in their home *kebele, and* were either currently on TB treatment or had received negative smear microscopy results. Individuals were screened for TB using a standard protocol (of Federal Ministry of Health of Ethiopia), and those with symptoms were asked to submit sputum samples. Sputum samples were collected at no cost to the patient. TB treatment was provided according to NTP guidelines.

The methods for this study were chosen to allow exploration of some of the complex phenomena relating to social, cultural and environmental circumstances that were not explained by quantitative data routinely collected through the intervention and to complement those data [27,28]. In-depth interviews (IDIs) were conducted to capture the perspectives and experiences of different participant groups [29]. We selected participants from the intervention project registers across six districts to ensure representation of the range and diversity in the region. Participants were from 18 to 65 years old. More women and people from lower socio-economic groups were identified (by HEWs) and purposively recruited as they were specifically targeted by the intervention packages. We recruited 36 participants for interview, 12 of whom had received a smear negative result, saturation (no new themes arising) occurred within this sample size. Data collection took place between May 2011 and February 2012.

A local interviewer (GA) and an assistant with experience of conducting qualitative research on TB conducted the interviews in Amharic or Sidaminigna. An interpreter was used for interviews conducted in Sidaminigna. Data were recorded, transcribed to Amharic and translated to English. A thematic framework was developed - guided by the different steps symptomatic individuals take within the intervention packages (from treatment seeking to diagnosis) by two of the study team’s social scientists (OT and FM). This was shared with six study researchers who contributed to the framework and collaboratively adapted and refined it according to principles of the framework approach [30]. Coding and data analysis was done using NVivo software (v.9).

**Figure 1:**
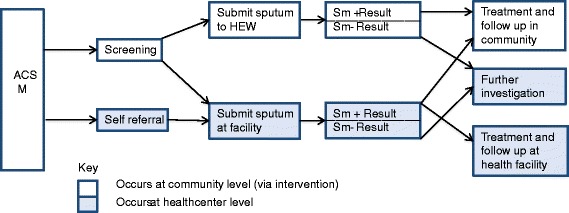
**Community perspectives pathway.**

Interviews explored decisions and choices; perceptions, experiences and opinions of the intervention and barriers to health care both during and prior to the intervention. Participants were asked to describe the path they took to TB services from the onset of illness so that the diversity of their experiences could be understood; the ways that they were made aware of TB screening and diagnostic services; how they felt about the process of producing sputum (at home or at the health post) and their results. In the case of smear negatives, their reaction and response to their inconclusive diagnosis was also sought. A simplified figure illustrating the steps patients took through the community based intervention packages was developed (Figure 1), the steps were confirmed in the analytic process and are used to structure the results. In reality, the participant testimonies created an array of pathways more complex than the linear process shown.

Illustrative quotations are annotated according to TB status: Patients diagnosed as having TB (Pt) or symptomatic person with negative smear-microscopy (Neg) and sex.

The Ministry of Health of Ethiopia and the Southern Region Health Bureau provided approval to the implementation of the project and conduct the study. Ethical approval was obtained from the Liverpool School of Tropical Medicine, UK (protocols 10.69 and 11.36LT). Verbal consent was obtained from participants (as many were illiterate) and observed and signed by an independent witness selected from the community.

## Results

### Participant characteristics

Few participants had regular income and most were engaged in subsistence agriculture, many had difficulty procuring sufficient food for their families. Most had no education, although younger participants were more likely to have attended primary school. Family size was generally large (usually more than four children, up to twelve children).

### Patient journey within the intervention packages

Figure 1 illustrates the steps that participants undertook in obtaining diagnosis and treatment, and contrasts it with the passive case finding approach. Variations (depicted by the arrows in diagram) did occur, with the routes taken dependent on factors including gender, age, economic circumstances, access to information and knowledge, family support structures, proximity to health services, clinical presentation and severity of disease and contact with HEWs and CHPs or other service providers.

### Pre-diagnosis

Patients diagnosed through the intervention package had usually waited a long time (several months or more) before diagnosis. Participants described initially seeking treatment from local drug sellers or using traditional medicines, other participants said they had been misdiagnosed when attending health facilities. Some had been ill for extremely long periods (sometimes several years). One important reason stated for delays was inaccessibility of heath facilities. This was mentioned by all types of participants, but particularly by women, frail or elderly participants.

*“Before there was no health institution available nearby… Five years have elapsed since I contracted this disease… I didn’t do anything, I bought medicine from the local dealer…I was almost dying.”* (Pt, woman, 35 yrs)

*“On foot, it takes 2 hours from here to the health centre, but for me it takes 3 and half hours.”* (Pt, Man 45 yrs)

*“Even if I feel like going (to health facility), I don’t have money… I’m poor*.” (Neg, woman, 45 yrs)

For those whose TB symptoms started shortly before or during the intervention reaching a diagnosis was generally simpler and started as a result of the intervention’s awareness creation.

### ACSM, screening and testing

Patients described how they were identified by CHPs or HEWs during house-to-house visits or at the health post. Others had participated in ACSM activities and then decided to attend a health post for examination or were guided by friends or neighbours who had attended ASCM; they described being informed about TB symptoms and the availability of free TB services within their *kebele*. Many people had visited the health post immediately as a result of ACSM activities conducted at churches and public meeting places.

*“Previously I was suffering from coughing. I was in my home without any treatment. But due to the awareness creation for those who have a cough I came to the health post.”* (Pt, Man, 18 yrs)

*“During coffee-ceremonies, whenever I hear someone coughing, I advise them to go to health post to take examination.”* (Pt, Woman, 45 yrs)

*“Health promoters were making announcements, through a megaphone, that anyone who has been coughing for more than two weeks must be examined. I happened to hear this announcement and was examined.”* (Neg, Man, 24 yrs)

Interviewees recognized the novelty and benefit of the intervention, and how in the past they would have spent time and incurred financial costs travelling to more distant health centres. Several respondents mentioned that previously ‘*people were dying at home’* but with the intervention ‘*everyone had heard’* that the service was available in the *kebele*.

*“I did not expend any money as the CHP saw me during a house-to-house visits and advised me to undergo examination.”* (Neg, Woman)

*“The HEW came when I was very sick. Their work of the house-to-house visit is good*.” (Pt, Woman, 22 yrs)

Following screening, symptomatic cases submitted sputum samples at home or in the health post. Participants indicated the intervention diagnostic processes was acceptable to them, being frequently described as ‘*easy’* or that they were ‘*happy’* about the service, this was the case whether they self-presented, or were screened at home or the community. There were no negative testimonies about screening or diagnostic procedures. However they described feelings of ‘*tension’* or ‘*fear’* whilst waiting the result to come back from the laboratory. Results generally were received between three and seven days later.

### Receiving results

Patients, many of whom had been very sick prior to diagnosis, had often felt relieved to know that they had TB and that treatment was available within their immediate locality. Some patients, mainly women, made clear contrasts to the past where diagnosis and treatment (which involved travel) was inaccessible because of the prohibitive costs and opportunity costs of seeking care at a more distant health centre. Illustrative quotations include:

*“I was very happy because my disease was found before my death.”* (Pt, Woman)

*“I felt very happy. My disease might have been killing me. Now I hope I will be cured of the disease.”* (Pt, Woman, 50 yrs)

*“I clapped my hands in delight and took my drugs and I prayed to God for his help. Because I am poor, I [previously] could not get treatment due to [lack of] money.”* (Pt, Woman, 45 yrs)

However not all respondents felt the sense of relief, with a few indicating they had suffered deeply on hearing they had TB. Emotions described included depression, fear of death, suicidal thoughts, fear of discrimination or being thought of as being HIV positive.

*“I was very depressed. I thought I was dying. Since I have children I was thinking of them.”* (Pt, Woman, 35 yrs)

Smear negative cases were offered broad-spectrum antibiotics and those without clinical improvement were requested to resubmit sputum samples and/or referred for X-ray, as recommended by the NTP. The transport and X-ray costs for referred patients who could not afford the expenses were covered by the project. A minority of respondents were extremely pleased not to have TB, but on the whole undiagnosed respondents were disappointed by their negative results. Because their health problem was still unresolved, they felt uncertain about their futures and fears of further costs or opportunity costs relating to diagnosis and treatment from further afield.

*“I feel much sorrow. I gave them my sputum and they said I was negative but still I feel pain inside, I don’t eat, I have become very thin, therefore I am not happy about the result.”* (Neg, Woman, 20 yrs)

*“What makes me unhappy is that no treatment is given to me on the result. Then I left here and bought tablets from other place.”* (Neg, Woman, 45 yrs)

*“They told me that I’m negative, they said you don’t have TB, but I’m still coughing. I’m praying to my lord because he knows everything.”* (Neg, Man, 60 yrs)

### Treatment and perceived impact of the intervention

Most attitudes towards the treatment itself were positive, although the perceived stigma attached to TB remained a concern for some (particularly women). Patients were willing to go to the health post, health centre or receive their drugs at home and no participants complained about treatment observation.

*“I am taking my drugs properly… Since I started taking treatment, all my problems are reduced.”* (Pt, Woman, 45 yrs)

*“I didn’t tell anyone [my result]. If I told them they wouldn’t have a good attitude. I didn’t want to tell them. I only told my mother*.” (Pt, Woman)

A considerable challenge that remained for many patients was their inability to support themselves financially and in particular obtaining adequate food during their recovery. For example:

*“The medicine alone makes you feel bad [side effects] unless you take food. They informed me of this, but I told them that I am not able to get enough food*.” (Pt, Woman)

Opinions about the diagnosis and treatment process were often voiced in terms of contrast to the pre-intervention period. On the whole participants spoke extremely positively about the availability of TB diagnosis and care in their home communities. For most, there had previously been no nearby health facility to manage TB so people had needed to go to distant healthcare providers. Those who had received TB services through the intervention were often enthusiastic about their desire to advocate for others to get diagnosis and treatment now that it had become available in the community.

### Recalled experiences from before the intervention

*“People with sufficient money went for treatment. But the people who didn’t stayed at home and died.”* (Pt, Woman)

### Experiences since the intervention

*“Society has information about the service, especially those who were sick like me have heard. There are also some who have not heard, if so I always tell them at any opportunity.”* (Pt, Woman)

Most respondents had a high opinion of the service given ‘*at no cost*’ in a ‘*convenient’* manner. The free services available within the *kebele* was a clear advantage noted by most respondents, including some who felt that they would not have been able to access TB services had they needed to travel to the health centre.

“*My family would not be able to afford the services I have been given, but it was covered by the project. If this money was to be paid by my family they would have to sell their cows.*” (Pt, Woman)

*“Since most of the people in this community are poor, it is very good to get examination nearby.”* (Neg, Man, 24 yrs)

*This is very important for the community. I had been sick for a long time and I couldn’t get to health facilities because of my problems. If I had stayed without treatment, I might have died.” (Pt, Woman, 50* yrs*)*

*“I am not able to go to far places to be treated because I don’t have money for transportation and food. Here in my community, without going to the health centre, I am getting treatment.”* (Pt, Woman)

## Discussion

TB control requires highly effective and affordable approaches that increase the number of cases in low income and remotely located populations. There is increasing effort to assess whether innovative intervention packages that deliver services close to the community are effective. A crucial characteristic to the success of these packages is their acceptability by potential users and the wider community, with recent WHO guidelines suggesting that ethical considerations and human rights issues also be taken into account. However, meaningful evidence for this aspect of interventions is often lacking.

The implementation of the package provided innovative TB diagnosis and treatment at household and community level and created an opportunity to evaluate its acceptability by the community. The intervention package was considered beneficial by all TB patients in this qualitative study, regardless of age or sex. Patients received information about their medical problems and of the availability of treatment for their condition. If the smear was negative they were advised to repeat the smear examinations and to seek medical attention at higher levels of the health system. Many patients seemed to prefer being diagnosed with TB than having a negative smear microscopy result. The latter meant uncertainty: they remained without diagnosis and further time and money to travel to health facilities for further examinations was anticipated. This group appeared more likely to be disappointed because a conclusive diagnosis could not be guaranteed, nor their expectations for treatment. High costs and long distances to health centres at different stages of health seeking had previously precluded many from receiving diagnostic services and many participants stated that prior to the intervention people with TB often died at home or remained without care. Receiving comprehensive TB services at a primary care level from within the community was highly appreciated and considered easy and acceptable. Community members willingly presented themselves for screening or accepted screening in their homes. This feature of the intervention can be considered a particular advantage for poor individuals, women and those who were very frail because of their challenges, costs and opportunity costs in accessing care.

The poor are more vulnerable to TB, furthermore they experience multiple barriers in accessing diagnostic services. Once diagnosed, the costs relating to obtaining treatment are also more pronounced for poorer populations, despite being free at the point of delivery, and they have worse treatment outcomes [3,4,31-34]. Community embedded ACSM, and bringing services close to communities, (so participants did not need to travel to diagnostic and treatment units previously only provided in hospitals and health centres) was perceived as increasing the accessibility of services by these poorest groups. A study from Northern Ethiopia suggests that the majority of patients do believe TB to be curable [35]. In contrast we found respondents who had believed the only outcome of TB was death, this may be due to the inaccessibility of services previously. TB in this context is often associated with cancer and HIV, and therefore respondents welcomed the opportunity to learn that TB is curable and that the treatment was being made very accessible. Our study also attempted to explore the potential negative implications of screening and highlighted that patients with chronic cough with negative smear-result often need further examinations and tests. Individuals with initial negative smear results in this study were revisited and there was an increase in the number of TB diagnoses as a result of either repeated smears the use of GeneXpert or a clinical diagnosis based on clinical findings and x-ray findings. We are aware of the additional needs for patients with unconfirmed TB and those who have a health problem that is not resolved by this package (asthma, COPD and so on). The intervention was only targeted to identify TB and patients with other chronic respiratory diseases were referred to services beyond the scope of the package described here. Much more research is needed to find solutions for this group of patients, as in fact they are the majority of patients identified by the package.

TB is reported in more men than women worldwide. When compared to passive case finding in Ethiopia, the package improved TB case detection particularly among women, with more women than men being screened and diagnosed with TB at community level [36]. For this reason, we purposively interviewed more women participants who reported that the intervention addressed some of the barriers faced through reducing delay, improving access, and supporting diagnosis. We were also interested in the perspectives of elderly people due to their reduced mobility and access to services, however this should not detract from recent evidence suggests that the TB burden in Ethiopia is greatest in the 15–34 age range [18].

From a patient perspective, our findings contrast the convenience of community-based service against facility-based TB services. These are often protracted and have a high risk of patient drop out. By drawing on the strength of the HEP, providing ACSM and then free services close to home, this intervention appears to offer some protection against factors inhibiting the most vulnerable groups from equitable access to services (poverty, inability to travel to health centres, gender, poor knowledge). Indeed most participants were relieved to receive a smear-positive TB diagnosis, because treatment was available within the community without having to incur additional costs. Those who were found smear negative were concerned about the next steps needed and for having to continue the treatment-seeking journey. Costs and logistics to access further screening tests, such as X-rays, were therefore introduced by the project. The majority of symptomatic individuals will have negative smear results and need further follow up and tests including repeated testing of their sputum samples at the community level. Supporting these patients to access a wider range of diagnostic tests for chronic respiratory problems, and making sure they are aware of the treatment options is an important consideration for community-based TB interventions.

Poor rural populations have low resilience to absorb healthcare costs. The intervention package in this setting has the potential to strengthen universal coverage and access to TB services in underserved areas, minimising costs and opportunity costs. This is critical to maximise equitable provision of services by making them universally accessible. Discussions on universal coverage often emphasises cost-effectiveness whilst losing sight of its central tenet of health for all [37]. Yet universal coverage does not ensure equity nor guarantee universal access to services [38]; it can only be considered pro-poor if poor people can *reach* services and *wish to do so*. Understanding why some people or communities do not receive adequate health services (effective coverage) or do not wish to access them (acceptability), requires analysing each component in the delivery of services and ensuring the views of those currently disenfranchised are elicited and empowered to ensure ‘reaching the unreached’ [39]. In rural Sidama, the qualitative experiences highlight how these populations can be reached by using a strong ACSM strategy which is linked with decentralization of diagnostic and treatment services embedded at the community level.

We have attempted to capture the voices of community members, and have described their support of the intervention. Several methodological constraints need to be considered: Interviews in Sidamigna were translated to Amharic and then all transcripts were translated to English, creating potential for loss of nuance and meaning. There was a risk that participants felt they should respond positively about the intervention so interviewers were specifically requested to pay attention to probing that would capture a full range of experiences, however triangulation through use of supplementary methods would have given the data further reliability. Stigma is highly prevalent for this disease and participants may have wished to please the interviewers. For this reason, the potential of the intervention to reduce stigma needs to be further explored. We ensured anonymity and encouraged open critical discussion but were unable to recruit participants who had refused screening as refusal was not reported; it is possible that some symptomatic individuals may have avoided screening or found the intervention and process less acceptable whose opinions are not represented. Although the main findings of the acceptability of community based diagnostic and treatment services for poor rural communities are likely to be transferable throughout Ethiopia, this is a culturally, geographically and linguistically diverse country and these findings are embedded within the particularities of the Zone.

The project benefited from partnership with the Ministry of Health of Ethiopia in implementing the HEP and focusing on TB prevention and control activities, with its investment in community-based structures and the development of CHWs and CHPs cadres. Although this initiative provides an exciting model for supporting close-to-community services, approaches need to be developed for settings where community level health-workers are unavailable. Further innovation and experience sharing of bringing TB services closer to communities is therefore required to support universal access.

## Conclusions

Rural populations with high levels of poverty - particularly women - are at high risk of unmet health needs and undiagnosed TB cases [5,20,33]. HEWs are strategically placed to strengthen accessibility within these populations. Ensuring universal access to health services requires innovations to enhance access to diagnostic and treatment services that are sustainable, acceptable and patient friendly. Increased health services coverage should lead to better population health and the largest gains *should* accrue to poorer people and those in remote or rural settings [40]. There is need to ensure that innovative approaches for case finding and treatment incorporate the experiences of the individuals targeted to inform further programme development. Embedding services within communities was valued by poor and rural communities with limited access to health facilities. In the Ethiopian context this approach can therefore be advocated as part of a strategy to strengthen surveillance and facilitate early diagnosis and treatment.
